# Nurses as Stewardesses

**Published:** 1889-03-02

**Authors:** 


### Editor's Letter-Box.

[Our Correspondents are reminded that prolixity is a great bar to publication, and that brevity of style and conciseness of statement greatly facilitate early insertion.]

### NURSES AS STEWARDESSES.

Sir,—You and your correspondents have been discussing the question of "Trained Nurses as Stewardesses on board Ships." To a medical mind it is simply astonishing that every passenger ship has not been provided with a trained nurse as stewardess, from the very beginning of the period when the trained nurse became a recognised institution. All passenger ships of any importance carry qualified medical men; yet the kind of medical work required is much more nearly allied to nursing than to "physicking." Especially is this the case with female passengers. What can be the reason why this obvious improvement has not been effected? Is it that the directors have not thought of the matter? Is it that the present stewardesses are cheaper than trained nurses would be? That can hardly be the case. Is it that nurses are unwilling to act as stewardesses? That is very unlikely. Lady passengers should insist upon trained nurses being provided for every ship in which they travel. The case is so clear, the argument so unanswerable, the comfort to passengers would be so great, the saving of health would be so decided, that it is impossible for an outsider to understand why the change has not been effected almost as soon as proposed.—I am, &c. M. D.

